# DNA Ligase IV Prevents Replication Fork Stalling and Promotes Cellular Proliferation in Triple Negative Breast Cancer

**DOI:** 10.1155/2019/9170341

**Published:** 2019-01-31

**Authors:** Rashmi R. Joshi, Sk Imran Ali, Amanda K. Ashley

**Affiliations:** Department Chemistry and Biochemistry, New Mexico State University, Las Cruces, NM 88003, USA

## Abstract

DNA damage is a hallmark of cancer, and mutation and misregulation of proteins that maintain genomic fidelity are associated with the development of multiple cancers. DNA double strand breaks are arguably considered the most deleterious type of DNA damage. The nonhomologous end-joining (NHEJ) pathway is one mechanism to repair DNA double strand breaks, and proteins involved in NHEJ may also regulate DNA replication. We previously established that DNA-PKcs, a NHEJ protein, promotes genomic stability and cell viability following cellular exposure to replication stress; we wanted to discern whether another NHEJ protein, DNA ligase IV (Lig4), shares this phenotype. Our investigations focused on triple negative breast cancer cells, as, compared to nonbasal breast cancer,* LIG4* is frequently amplified, and an increased gene dose is associated with higher Lig4 expression. We depleted Lig4 using siRNA and confirmed our knockdown by qPCR and western blotting. Cell survival diminished with Lig4 depletion alone, and this was associated with increased replication fork stalling. Checkpoint protein Chk1 activation and dephosphorylation were unchanged in Lig4-depleted cells. Lig4 depletion resulted in sustained DNA-PKcs phosphorylation following hydroxyurea exposure. Understanding the effect of Lig4 on genomic replication and the replication stress response will clarify the biological ramifications of inhibiting Lig4 activity. In addition, Lig4 is an attractive clinical target for directing CRISPR/Cas9-mediated repair towards homology-directed repair and away from NHEJ, thus understanding of how diminishing Lig4 impacts cell biology is critical.

## 1. Introduction

Replete DNA damage is a hallmark of cancer and aberrant function of the DNA damage response proteins is known to be associated with many cancer subtypes [1]. Many chemotherapeutic drugs induce replication stress and resultant DNA damage; thus, the DNA repair pathways regulate the cellular response to chemotherapeutic intervention and can influence development of drug resistance. Arguably the most deleterious lesions, DNA double strand breaks (DSB), can arise from damage induced by both endogenous and exogenous sources, including but not limited to ionizing radiation, genotoxic chemicals, replication errors, and generation of reactive oxygen and nitrogen species. In mammals, DSB are predominantly repaired by two major pathways: homologous recombination (HR) and nonhomologous end-joining (NHEJ, for a review, please see [2, 3]). NHEJ is the major DSB repair pathway in mammalian cells [4] and, in contrast to HR, is not dependent upon the availability of a homologous DNA template for repair. HR is considered to be “error free” in comparison to NHEJ due to template usage and is the primary repair pathway responsible for DNA replication fork restart during cellular division.

In NHEJ-mediated repair, DSB are recognized by the Ku70/Ku80 dimer, which recruits DNA-PKcs, Artemis, and DNA ligase IV (Lig4) with XRCC4 [2]. Lig4 catalyzes the phosphodiester bond formation during the final step of NHEJ [2]. The C-terminal domain of Lig4 contains two tandemly arrayed BRCT domains flanking the XRCC4-interacting domain, which facilitates its interaction with its binding partner XRCC4 [5, 6]. End processing can result in small insertions or deletions at the break site, so NHEJ is considered a more “error-prone” repair pathway compared to HR, though it repairs the majority of cellular DSB [4]. While DNA ligases I and III participate in other forms of DNA metabolism, the activity of Lig4 is solely associated with NHEJ [2]. While “classical” NHEJ, the major DSB repair pathway in cells, is associated with Lig4, repair via alternative end-joining pathways may be mediated by DNA ligases I or III [7–9], though these are not as frequently utilized.

In addition to DSB repair, the role of HR in mitigating DNA replication stress is well known [11]; however, whether NHEJ proteins may also modulate cellular recovery was unclear. We demonstrated cells lacking an early acting NHEJ protein, DNA-PKcs, restarted DNA replication quicker than wild type due to an inability to fully activate/phosphorylate cellular proteins involved in the DNA damage response, including RPA32, Chk1, and *γ*H2AX [10, 12, 13]. To discern whether aberrant replication restart was due to dysfunction of NHEJ, we knocked down Lig4 to determine whether it has a similar role in regulating unstressed and/or compromised DNA replication. We investigated whether Lig4 might be a putative therapeutic target in a disease where HR is often compromised, namely, triple negative breast cancer (TNBC).

TNBC is a clinical subtype of breast cancer lacking nuclear estrogen receptor and progesterone receptor and does not overexpress the growth factor receptor HER2. While not all basal cancers are TNBC and vice versa, basal-like TNBC is associated with lower survival [14] and is the most common classification of TNBC, accounting for approximately 70-80% of patients [15]. Patients with TNBC suffer from poor prognosis [16], shorter relapse-free survival, higher risk for recurrent disease [17], and an increased risk of dying as a consequence of tumor relapse compared to other breast cancer types [18]. While patients with TNBC face an overall poor prognosis, after five years, the risk for recurrence and tumor-related death diminish to approximately the same level as the non-TNBC [19]. This suggests TNBC patient survival is highly dependent upon preventing recurrence and chemotherapy resistance. Approximately 80% of patients with hereditary mutations in the HR protein BRCA1 present with TNBC, and an additional 61-69% are characterized by “*BRCA*ness” compared to 39% of other breast cancer subtypes [20]. As such, TNBC patients frequently have compromised HR and this may cause patients with TNBC to be particularly reliant upon NHEJ-mediated DNA repair. Therefore, we sought to understand the potential therapeutic value of targeting Lig4 in TNBC and the cellular ramifications of decreasing Lig4. Increased NHEJ contributes to developing resistance to chemotherapy in TNBC [21], so targeting this pathway via interfering with Lig4 may provide a novel treatment option in TNBC.

## 2. Materials and Methods

### 2.1. TCGA Analysis

The TCGA database was interrogated using cBioPortal [22, 23]. Lig4 was assessed in all breast cancers profiled in the METABRIC dataset, the basal only subset, and all breast cancer types excluding the basal subtype [24]. The OncoPrint, indicating observed genomic alterations, is presented. cBioPortal was used to obtain* LIG4* mRNA expression z-scores generated using an Illumina Human v3 microarray to permit a comparison of genomic alterations (deep deletion, shallow deletion, diploid, gain, or amplification) with mRNA expression levels. Definitions of these terms are available at the cBioPortal website: http://www.cbioportal.org/faq. Database was accessed for data used in this project last in August 2018.

### 2.2. Cell Culture

Unless otherwise indicated, all laboratory chemicals were purchased from Sigma Aldrich (St. Louis, MO). Human breast cell lines BT549 or MDA-MB231 were obtained from ATCC (Manassas, VA) and grown under 5% CO_2_ at 37°C at 100% humidity. Base medium (Life Technologies; Carlsbad, CA) was as follows: BT549: DMEM/F12, MDA-MB231: DMEM, and each was supplemented with 10% FBS and 100 units/mL penicillin/streptomycin (Life Technologies). Cells were transfected with siRNA targeting Lig4 (Qiagen; Hilden, Germany, Cat# SI00036022) or a nontargeting control (NT; Qiagen AllStars Negative Control) at 10 nM using RNAiMax (Life Technologies) for indicated times. Knockdown of Lig4 was confirmed using western blotting and/or qPCR for each experiment.

### 2.3. qPCR

RNA was isolated along with on-column genomic DNA digestion from cells using RNeasy Mini Kit (Qiagen) as per manufacturer's instructions. RNA was quantified using NanoDrop 2000 spectrophotometer (Fisher Scientific, Hampton, NH). RNA samples were stored at −80°C until further analysis. cDNA was synthesized with 1* μ*g RNA using the iScript cDNA Synthesis Kit (BioRad, Hercules, CA) according to manufacturer's instructions and then diluted with nuclease free water to a final concentration of 10 ng/*μ*L. qPCR was performed with CFX Connect Real-Time PCR Detection System (BioRad) using iTaq Universal SYBR Green Supermix (BioRad). Primer sequences used are as follows: Lig4: 5′ TGC TGC TGA GTT GCA TAA TGT 3′, 5′ AGC AGC TAG CAT TGG TTT TGA 3′ (designed against X83441.1); GAPDH: 5′ ACA GTC AGC CGC ATC TTC TT 3′, 5′ ACG ACC AAA TCC GTT GAC TC 3′ (designed against NM_002046.7). Prior to use in qPCR, we assured our primers sets did not produce primer dimers and primer efficiencies were assessed to ensure equal amplification across varying starting levels of template. Forward and reverse primers were used at a final concentration of 0.5  *μ*M with 50 ng cDNA. The qPCR conditions were 95°C for 3* *min, 40 cycles of 95°C (30s), 60°C (30* *s), and 72°C (15* *s) followed by a melt curve. Cq values of target were normalized to Cq values of GAPDH via the 2^−ΔΔCq^ method [25]. The amplicons were sequenced to confirm specificity (data not shown).

### 2.4. DNA Fiber Assay

BT549 cells were transfected with siRNA targeting Lig4 or NT as described. Cells were pulsed with the thymidine analog 5-iodo-2′-deoxyuridine (IdU; 20 *μ*M) in complete growth medium and incubated for 15 min at 37°C. Media was removed and cells were washed with PBS three times followed by a brief wash with thymidine (100 *μ*M). Cells were then exposed to fresh media containing the second thymidine analog 5-chloro-2′-deoxyuridine (CldU; 20 *μ*M) for 20 min at 37°C. Alternatively, following the original IdU pulse, cells were then treated with either hydroxyurea (HU, 10 mM) or vehicle (PBS) for an hour in complete growth medium at 37°C. Medium was removed and cells were washed with PBS and were then pulsed with CldU (20 *μ*M) for 30 min at 37°C. After exposure to both thymidine analogs, cells were subjected to the DNA fiber assay [10]. Cells were washed with PBS and harvested by trypsinization and then resuspended in PBS. Two thousand cells were transferred to a positively charged microscope slide (Superfrost Plus, Daigger, Vernon Hills, IL), lysed with lysis buffer (0.5% SDS, 200 mM Tris pH 7.4, 50 mM EDTA), and incubated at room temperature for 3 min. Slides were dropped at an angle to allow DNA fibers to spread across the length of the slide, air-dried for 8 min, and fixed in freshly prepared 3:1 methanol:acetic acid for 5 min. Slides were again air-dried for 8 min and stored in 70% ethanol at 4°C overnight. The next day, slides were incubated in 100% methanol for 5 min and washed with PBS. Deproteinization was carried out by incubating in 2.5 N HCl at 37°C for 1 hr followed by blocking with 5% bovine serum albumin at 37°C for 15 min. IdU and CldU were detected sequentially using fluorescently labelled secondary antibodies, all antibody incubations were conducted for 45 min at 37°C. Briefly IdU was detected using mouse anti-BrdU antibody (Fisher Scientific) followed by secondary goat anti-mouse Alexa 568 (Life Technologies). CldU was detected using rat anti-CldU (Accurate Chemicals, Tempe, AZ) and secondary donkey anti-rat Alexa 488 (Life Technologies). All antibody dilutions were prepared in 0.5% bovine serum albumin and washes were performed using PBS. Slides were mounted using ProLong™ Gold Antifade Mountant (Fisher Scientific). DNA fibers were visualized and captured using a Zeiss Axioimager Z1 motorized microscope equipped with a XCite LED epifluorescence light source, Apotome 2 structured illumination module and Axiocam 506 CCD camera. Replication forks labelled only red (IdU positive only) were defined as stalled forks, those labelled only green (CldU positive only) were defined as new forks and those labelled both red and green (IdU+CldU) were defined as restarted forks. Images were scored by the person who was blinded to the sample identity. Data was analyzed as stalled or restarted or new forks and determined as a percent of the total number of forks scored per image. Mean, standard deviation was calculated for each treatment condition from all the scored images per condition.

### 2.5. Western Blotting

BT549 cells were grown in 60 mm culture plates at a concentration of 500,000 cells per dish. At 24 hr after transfection, cells were treated with four mechanistically different replication toxins (doxorubicin, DOX, 1 *μ*M; etoposide, ETOP, 10 *μ*M; HU, 2 mM; camptothecin, CPT, 0.2 *μ*M) or vehicle (DMSO) and protein was extracted after 24 hr of treatment. For pChk1 and pChk2 experiments BT549 cells were treated with 10 mM HU or vehicle (PBS) for 1 hr or 24 hr and protein was harvested immediately after treatment. For Chk1 and Chk2 dephosphorylation experiments, BT549 cells were treated with 10 mM HU or PBS for 1 hr and released for indicated times followed by protein harvest. For pDNA-PKcs S2056 experiments, BT549 cells were treated with 10 mM HU or PBS for 1 hr or 24 hr and released for 2 hr in complete cell growth media followed by protein harvest. Whole cell lysates were obtained using RIPA buffer [50* * mM Tris (pH 7.4), 2* * mM EDTA, 150 * *mM NaCl, 0.1% SDS, 1.0% Triton X-100) supplemented with Halt phosphatase and protease inhibitor cocktails (Fisher Scientific)]. Protein was quantified using Pierce BCA Protein Assay (Fisher Scientific) per manufacturer's instructions. Protein (15 or 30 *μ*g) was subjected to SDS-PAGE and transferred to PVDF membrane. Membranes were blocked with either 5% milk or 5% BSA in 1X tris-buffered saline with 0.1% Tween-20 (TBS-T) at room temperature for one hour. The primary antibody was diluted in the blocking solution and incubated overnight at 4°C. Membranes were washed in 1X TBS-T and appropriate HRP-conjugated secondary antibodies were applied in blocking buffer for one hour at room temperature. Blots were washed with TBS-T and Clarity Western ECL Substrate (BioRad Cat# 1705061) was applied followed by detecting the chemiluminescence using ChemiDoc MP Imaging System (BioRad). The following primary antibodies were used: Lig4, DNA-PKcs and pDNA-PKcs S2056 (Abcam, Cambridge, UK), *β*-actin and Vinculin (Santa Cruz Biotechnology, Santa Cruz, CA) and GAPDH, cleaved caspase 3, caspase 9, *β*-tubulin, pChk1-S317, pChk1-S345, total Chk1, pChk2-T68, or total Chk2 (Cell Signaling Technology, Danvers, MA). Secondary antibodies used were: HRP-conjugated goat anti-rabbit and goat anti-mouse (Jackson ImmunoResearch, West Grove, PA).

### 2.6. Cell Viability

Cells were grown overnight in 96-well white-walled plates (CoStar, Corning, NY) and subsequently transfected with siRNA targeting Lig4 or NT for 72 hr. CellTiter Glo luminescent cell viability assay (Promega, Madison, WI) was used to quantify viable cells per manufacturer's instructions 72 hr after transfection. To assess clonogenic survival, BT549 cells were transfected for 24 hr and then transfected cells were seeded onto 60 mm dishes at a seeding density of 500 cells per plate. Cells were allowed to attach for 4 hr and incubated at 37°C for 7-10 days with fresh media replenished every three days. Following incubation, cells were washed once with 1X PBS, followed by fixation in 4% paraformaldehyde for 15 min. Cells were then stained with 0.5% crystal violet in 20% methanol for 2 hr. Cells were washed with water, air-dried, and colonies with ≥ 50 cells were counted. Plating efficiency was calculated as PE = (# of colonies formed / # of cells added) *∗* 100 and surviving fraction (SF) was calculated as: SF = (PE Lig4 depleted cells/ PE NT cells) *∗* 100. For all assays, the experiments were repeated at least three times with three replicates within each experiment.

### 2.7. Immunofluorescence

Cells were grown on four-well chamber slides overnight and transfected with siRNA targeting Lig4 or a scrambled control (NT) for 24 hr. Cells were treated with PBS for 24 hr, fixed with 4% paraformaldehyde for 10 min, permeabilized in 0.1% Triton X-100 for 1 min, washed and then blocked in 1% bovine serum albumin for 30 min at room temperature. The primary antibody recognizing p-S139-H2AX (*γ*H2AX; Abcam) was applied in blocking solution for 1 hr at room temperature. Cells were washed with PBS and an Alexa Fluor 488-conjugated secondary antibody (Invitrogen) was applied in blocking solution for 1 hr at room temperature. Cells were washed and mounted in ProLong™ Gold Antifade Mountant containing DAPI (Fisher). Images were captured digitally with a Zeiss Axio imager microscope using both green and blue channels for *γ*H2AX and DAPI respectively. For each condition 10 images were captured. *γ*H2AX foci were quantified using ImageJ software with DAPI stained area defined as region of interest (ROI). Non-DAPI stained region was used to account for background fluorescence and was subtracted from each of the *γ*H2AX mean intensity signal values. Mean intensity signal of *γ*H2AX was normalized to the DAPI stained area per image.

### 2.8. Statistical Analysis

All statistical analysis was performed using GraphPad Prism (La Jolla, CA). A Chi-square analysis with Yates' correction was used to evaluate* LIG4 *genetic alterations by tumor molecular classification; the p value was determined using a two-sided Fisher's exact test. The impact of* LIG4* gene dose on mRNA expression level was evaluated via one-way ANOVA and a linear trend from left to right was assessed* post hoc*. Statistical analysis of cell growth over time was evaluated with a linear regression coupled with an extra sum-of-squares F test. Unless otherwise indicated, cells exposed to NT or Lig4 siRNA were compared within treatment using a two-tailed* t* test.

## 3. Results

### 3.1. Lig4 Depletion Decreases Cell Viability

**Figure 1:**
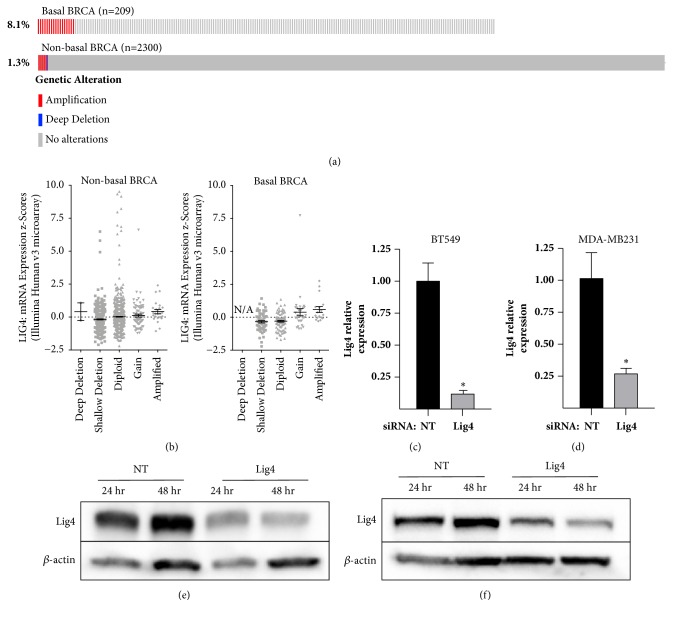
***LIG4* amplification in different breast cancer subtypes. **(a) Results were generated from cBioPortal analysis of the METABRIC breast cancer (BRCA) dataset (accessed April 2018).* LIG4* amplification is observed in 8.1% of patients with basal breast cancer (n=209), significantly more frequently compared to alterations, including amplification and deep deletions, observed in 1.3% of cases in data from breast cancer subtypes excluding basal (n=2300 patients; p<0.0001). (b) In all nonbasal BRCA patients genomic alterations in* LIG4 *were not associated with altered mRNA expression (n=2300, p=0.8348) whereas a positive association was detected in patients with basal BRCA (n=209, p<0.0001). Cells were transfected with a single construct siRNA targeting either Lig4 or scrambled control (NT) for 24 hr or 48 hr. Lig4 depletion was confirmed using qPCR (c, d; ∗p<0.05; data plotted are mean + SEM) and western blot (e: BT549, f: MDA-MB231).

**Figure 2:**
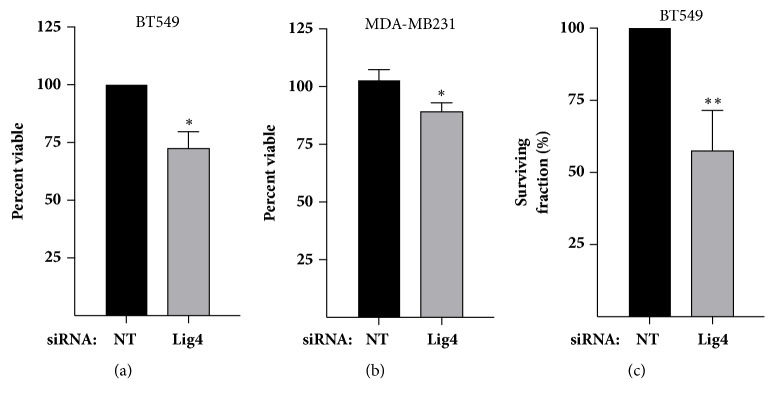
**Lig4 depletion reduced cellular viability and colony forming ability. **(a, b) Cell viability assessed using CellTiter Glo assay 72 hr after transfection with siRNA targeting Lig4 or scrambled control (NT; *∗*p<0.05). (c) BT549 cells depleted of Lig4 or scrambled control and subjected to clonogenic assay (*∗∗*p<0.01, n=3).

To assess the prevalence of* LIG4 *genetic alterations in basal versus non-basal breast cancer, we interrogated METABRIC cohort of breast cancer patients characterized in the Cancer Genome Atlas (TCGA) using cBioPortal. Amplifications in* LIG4* are observed in 1.8% of all breast cancers represented in the 2509 samples in the METABRIC dataset (accessed in August 2018). Of the 209 patients classified as basal, a significantly higher percentage, 8.1%, have* LIG4* amplifications compared to 1.3% observed in other molecular subtypes (Figure 1(a), p<0.0001). To determine whether these genomic alterations caused increased expression level, we assessed mRNA expression z-scores generated using an Illumina Human v3 microarray by TCGA (Figure 1(b)). For each data set, an ordinary one-way ANOVA was completed to interrogate whether the mean values for mRNA were different between individuals with genomic differences, namely via assessment of a linear trend from the most severe negative gene alteration (deep deletion for non-basal and shallow deletion for basal breast cancer patients, respectively) towards those harboring positive changes in gene dose: amplifications. A summary of these results is presented in Supplemental Table 1. A significant increase in* LIG4* expression was observed in the basal patients (slope=0.1735, p<0.0001) but not the non-basal cohort (slope=0.02979, p=0.8348), indicating the mRNA levels in basal patients increased with increased* LIG4 *gene dose (Figure 1(b)). To investigate the role of Lig4 in breast cancer, especially TNBC, we depleted Lig4 in basal breast cell lines and confirmed the knockdown using qPCR (Figures 1(c) and 1(d)) and western blot (Figures 1(e) and 1(f)). We assessed the effect of Lig4 depletion on cell viability after 72 hr after knockdown and observed a modest yet significant decrease in the cell viability (Figures 2(a) and 2(b)) due to decreasing cellular Lig4 levels alone. We employed the clonogenic assay to investigate the cellular reproductive death of Lig4 depleted cells [26]. The survival of TNBC cells with depleted Lig4 levels decreased approximately 50% compared to control cells (Figure 2(c)). Lig4 depletion did not markedly increase the cellular sensitivity to replication toxins (Supplemental Figure 1 and data not shown).

### 3.2. Lig4 Knockdown Decreases Cell Viability without Enhancing Apoptosis

**Figure 3:**
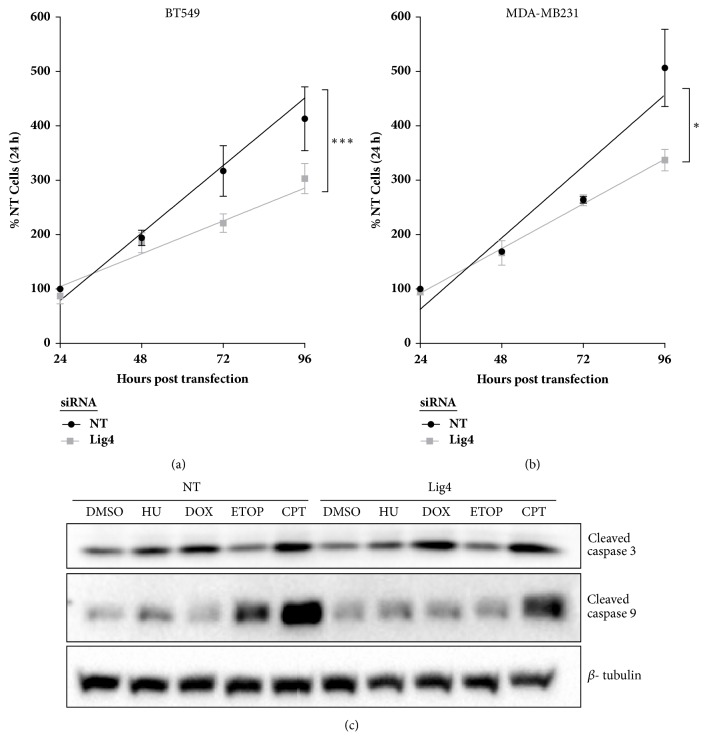
**Lig4 knockdown causes decline in cell numbers without enhancing apoptosis. **(a, b) Lig4 depleted or control (NT) cells were counted every 24 hr for up to 120 hr. Lines are the linear regression (*∗*p<0.05; *∗∗∗*p<0.001, n=3). (c) Levels of apoptotic markers cleaved caspase-3 and 9 were assessed using western blot in Lig4 depleted and control cells treated with either replication toxins (1 *μ*M doxorubicin, 2 mM hydroxyurea, 10 *μ*M etoposide, or 0.2 *μ*M camptothecin) or DMSO.

To determine whether the decrease in viability was a result of enhanced apoptosis we assessed apoptotic indicators. Lig4 depleted cells were treated with levels of replication toxins known to induce cell death (data not shown): CPT (0.2 *μ*M), HU (2 mM), ETOP (10 *μ*M), DOX (1 *μ*M) or with vehicle (DMSO) for 24h. Protein harvested was assessed using western blot for expression of the apoptotic indicators cleaved caspase 3 and caspase 9. The levels of apoptotic indicators were observed to be similar in both Lig4 depleted and control (NT) cells (Figure 3(c) and Supplemental Figures 2A and 2B) suggesting there was no robust enhancement of apoptosis associated with Lig4 depletion alone or following toxic insult. We employed a cell count assay to evaluate alterations in cell multiplication. Cells were counted every 24 hr for up to 120 hr following Lig4 knockdown. A depressed rate of cellular accumulation was observed in Lig4-depleted cells in comparison to the NT cells (Figures 3(a) and 3(b)), indicating a likely suppression of cell division.

### 3.3. Lig4 Prevents Replication Fork Stalling

**Figure 4:**
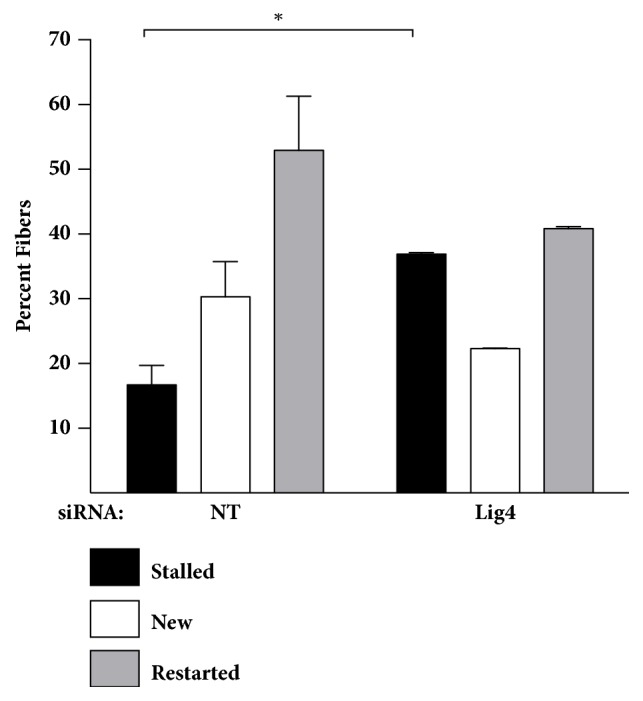
**Lig4 depletion alters basal levels of replication. **(a, b) BT549 cells were treated with siRNA targeting either Lig4 or scrambled control (NT) and DNA replication was assessed using the DNA fiber assay. Briefly, cells were sequentially pulsed with thymidine analogs IdU and CldU and the DNA fiber assay was conducted [10]. For each treatment, stalled, new and restarted replication forks were scored after blinding for treatment. Percent of stalled, new and restarted replication forks in Lig4 depleted cells were compared with respective NT control cells (*∗*p<0.05).

To discern whether decreased cell numbers following Lig4 depletion was caused by alterations in replication fork dynamics, we employed the DNA fiber assay. In Lig4 depleted cells, we observed an increase in the percentage of stalled replication forks in comparison to the control cells (Figure 4 and Supplemental Table 2). Cells exposed to PBS for an hour prior to receiving the second thymidine analog demonstrate similar results (Supplemental Figure 3A and Supplemental Table 3) In cells exposed to replication stress, as expected, a significant increase in number of stalled forks was observed with a concomitant decrease in the number of restarted forks in Lig4 depleted cells versus the NT cells (Supplemental Figure 3B and Supplemental Table 3). The differences observed are likely due to Lig4 depletion and not markedly enhanced by the addition of a replication toxin. Our results suggest that Lig4 depletion affects the basal level of replication in TNBC cells.

### 3.4. Lig4 Diminution Does Not Alter Checkpoint Activation

**Figure 5:**
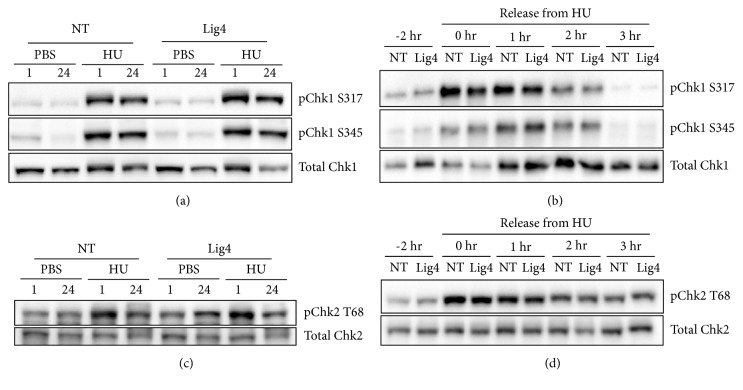
**Lig4 depletion does not increase Chk1 or Chk2 phosphorylation or dephosphorylation. **pChk1 (Ser317 or Ser345), pChk2 (Thr68), total Chk1, or total Chk2 were assessed via western blot. The experiment was repeated three times. (a, c) Lig4 depleted or scrambled control treated (NT) cells were treated with 10 mM hydroxyurea or PBS for 1 or 24 hr. (b, d) Lig4 depleted or NT cells were treated with 10 mM HU or PBS for 1 hr and released for indicated times.

One explanation for increased replication fork stalling could be an enhancement in DNA damage checkpoint signaling. Chk1 and Chk2 are checkpoint proteins activated via phosphorylation in response to DNA damage and known mediators of DNA damage signaling. We assessed Chk1 and Chk2 phosphorylation and dephosphorylation following replication stress. Chk1 is phosphorylated at two residues following DNA damage, S317 and S345 while Chk2 is phosphorylated at T68. Phosphorylated and total Chk1 were assessed using an immunoblot (Figure 5(a)) and remained similar between NT and Lig4 amongst treatments. Lig4 depleted and NT cells had similar levels of Chk1 dephosphorylation (Figure 5(b)). Chk2 phosphorylation and dephosphorylation at T68 were also unchanged by Lig4 depletion (Figures 5(c) and 5(d)). Together, these results suggest Lig4 depletion causes no impairments in activating or inactivating checkpoint signaling.

### 3.5. Lig4 Depletion Increases Basal *γ*H2AX Levels

**Figure 6:**
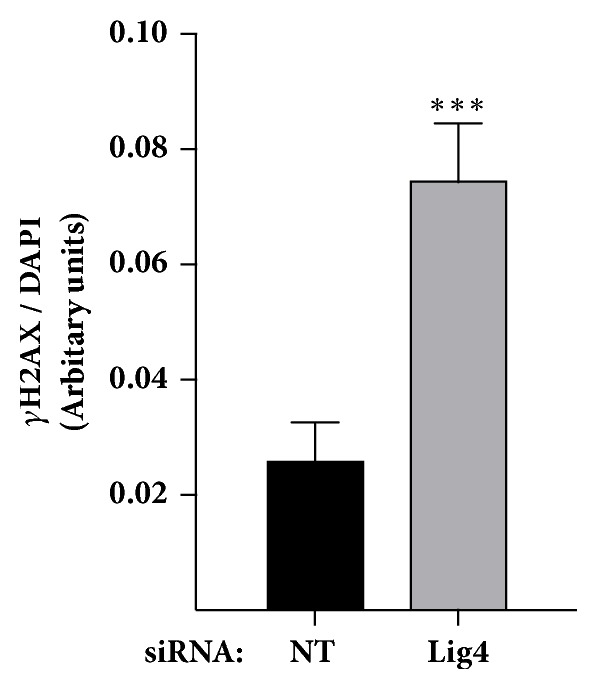
**Lig4 depletion alone increased persistence of DNA double strand breaks. **BT549 cells were transfected with siRNA targeting Lig4 or scrambled control (NT) followed by immunostaining for *γ*H2AX foci. *γ*H2AX foci were quantified using ImageJ software with DAPI stained area defined as region of interest per image and scored. Mean intensity signal of *γ*H2AX was normalized to the DAPI stained area per image (*∗∗∗* p<0.001).

We examined the possibility that unresolved persistent DSB caused by diminished Lig4 levels might impair cellular replication. H2AX is a histone variant phosphorylated at S139 (referred to as *γ*H2AX) in response to DSB [27]. To understand if Lig4 depletion caused an increase in DSB, we assessed the formation of *γ*H2AX foci in Lig4 depleted and control cells. TNBC cells transfected with siRNA targeting Lig4 or scrambled control were treated with PBS for 24 hr and *γ*H2AX was detected using immunofluorescence. We observed an increase in *γ*H2AX foci in Lig4 depleted cells (Figure 6 and Supplemental Table 4). Increased *γ*H2AX foci in Lig4 depleted cells is likely indicative of persistent spontaneously arisen unresolved DSB.

### 3.6. Lig4 Depletion Increases Phosphorylation of DNA-PKcs

**Figure 7:**
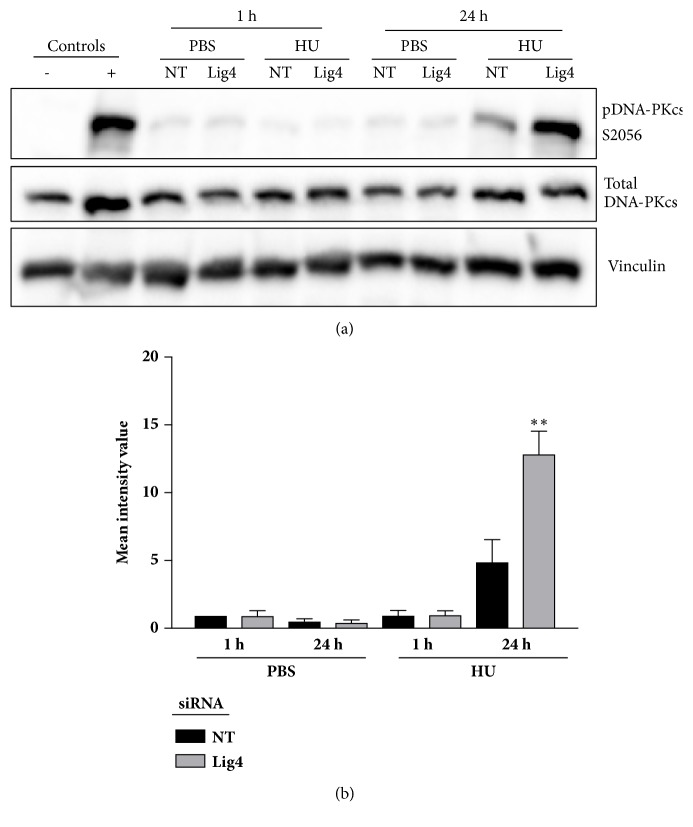
**Lig4 depletion causes increases in levels of pDNA-PKcs S2056. **(a) Lig4 depleted BT549 or scrambled control (NT) treated cells were treated with HU or PBS and protein isolated as outlined in the methods section. Phosphorylation of DNA-PKcs at Ser 2056 and total DNA-PKcs levels were assessed using immunoblot. (b) Densitometry analysis of three biological replicates. Loading control (vinculin) normalized mean intensity values of pDNA-PKcs S2056 per treatment were first normalized to total DNA-PKcs followed by normalizing to control cells treated with vehicle (1 h) values (*∗∗*p<0.01, n=3). Untransfected BT549 cell lysate served as the negative control and lysate from 10 Gy irradiated BT549 cells served as the positive control.

Aside from its well-established role as a key NHEJ protein, Lig4 likely promotes DNA-PKcs autophosphorylation at S2056 following ionizing radiation [28]. We evaluated the effect of Lig4 depletion on DNA-PKcs autophosphorylation at S2056. TNBC cells with or without Lig4 knockdown were treated with 10 mM HU or vehicle for 1 hr or 24 hr and released for 2h in complete growth medium after the indicated treatment times. pDNA-PKcs S2056 and total DNA-PKcs levels were assessed via western blot (Figure 7(a)). We found that Lig4 depleted cells showed increased levels of pDNA-PKcs S2056 with prolonged exposure to replication stress, which presumably had led to replication fork collapse and DSB (Figure 7(b)). Increased levels of pDNA-PKcs S2056 leads to limited end processing [29] while the existing DSB cannot be repaired due to the attenuation of Lig4, resulting in persistent DSB.

## 4. Conclusions

Lig4 is frequently amplified, resulting in elevated mRNA expression in patients with basal breast cancer. While not redundant terms, most TNBC is classified as basal breast cancer and vice versa. To assess the utility of targeting Lig4 in TNBC, a subtype with a poor prognosis and lacking in drug targets, we knocked down Lig4 levels in basal TNBC cell lines and observed a modest yet significant decrease in viability (Figures 2(a) and 2(b)) and decreased clonogenic survival (Figure 2(c)). Lig4 depleted cells proliferated comparatively slowly (Figures 3(a) and 3(b)) without a concomitant enhancement of apoptosis (Figure 3(c)). Further, we observed an increased percent of stalled DNA replication forks with an associated decrease in percent of new replication forks in Lig4 knockdown cells (Figure 4 and Supplementary Figure  3(a)). Taken together, these data indicate that Lig4 depletion affects basal levels of cellular replication.

We assessed the possible explanations for decreased TNBC replication due to Lig4 diminution including hyperactivation or failure to inactivate replication checkpoint signaling and/or an elevation in unrepaired DSB. Chk1 and Chk2 are serine/threonine protein kinases that phosphorylate other downstream DNA damage response effector proteins, ultimately promoting cell cycle arrest and blocking the G2/M transitioning [30, 31]. Chk1 and Chk2 activation following acute (1 h) or prolonged (24 h) exposure to HU was similar regardless of knockdown, indicating that Lig4 does not regulate their activation (Figures 5(a) and 5(c)). Similarly, a temporal investigation into Chk1 or Chk2 dephosphorylation following replication stress indicates that quelling of checkpoint activation is not dependent on Lig4 (Figures 5(b) and 5(d)). We assessed the formation of *γ*H2AX foci in Lig4 depleted and control cells, as this posttranslational modification is indicative of DSB [27]. We observed an increase in *γ*H2AX foci following Lig4 depletion (Figure 6). Addition of a replication toxin did not substantially enhance *γ*H2AX foci formation in Lig4 knockdown cells (data not shown). Collectively, our data suggest that increased *γ*H2AX foci in Lig4 depleted cells is likely due to persistent spontaneously arisen unresolved DSB and causes decreased rates of cellular proliferation.

Lig4 may contribute to DNA-PKcs autophosphorylation at S2056 following ionizing radiation [28], and this modification results in increased DNA-PKcs association with DNA at the DSB site [29]. DNA-PKcs is a very large protein (approximately 469 kDa) and its presence at the DSB restricts the access of end processing factors to the DNA damage site [32]. Since HR requires end processing for repair, these DSB would likely be preferentially repaired via the NHEJ pathway [32]. We evaluated the effect of Lig4 depletion on DNA-PKcs autophosphorylation at S2056. In our assay, Lig4 depletion increased levels of pDNA-PKcs S2056 with prolonged exposure to replication stress (Figures 7(a) and 7(b)) seemingly in contrast with previously published results indicating Lig4 promotes pDNA-PKcs S2056 [28]. However, Cottarel et al. demonstrate that though abolishment (knockout) of Lig4 does prevent phosphorylation of S2056, introduction of minimal amounts of Lig4, as present in our assays, is sufficient to drive DNA-PK phosphorylation [28]. We believe that this elevation in DNA-PKcs phosphorylation is indicative of unrepaired DNA damage due to Lig4 knockdown. In our results, the sustained exposure to HU results in replication fork collapse leading to an accumulation of DSB, which remains unrepaired due to Lig4 depletion preventing NHEJ-mediated repair coupled with an inability of DNA-PK to dissociate from broken ends to allow for HR-mediated repair [32]. We previously described the roles of DNA-PKcs in the cellular response to replication stress [10, 12], and they are distinct from what is observed with Lig4 depletion. This indicates that DNA-PKcs acts beyond its role in NHEJ in replication fork recovery, while Lig4's role in cell viability is consistent with its central role in NHEJ. Our results suggest Lig4 promotes cell survival through both resolution of spontaneous DNA damage via NHEJ and by dually ensuring dissociation of DNA-PKcs with DNA to permit end processing and HR-mediated repair.

The clinical ramifications of our research are multifaceted. These results indicate that the sensitivity to replication stress demonstrated by our lab and others when DNA-PKcs is deleted or inhibited is not redundant with its role as a NHEJ protein and dually suggests other mechanisms of sensitization. Further, our results demonstrate a modest decrease in cellular viability by decreasing Lig4 levels in TNBC, and yet decrements in Lig4 activity are associated with profound enhancements in sensitivity to ionizing radiation [33]. As such, inhibition of Lig4 may prove beneficial in combination with therapeutic application of the FDA-approved breast-specific stereotactic body radiotherapy device GammaPod, which delivers a high (8 Gy), but localized dose of radiation for breast cancer treatment [34]. We anticipate Lig4 depletion will enhance the efficaciousness of ionizing radiation in breast cancer treatment. The clinical ramifications of Lig4 inhibition are crucial in consideration of directing DSB repair towards HR versus NHEJ in the context of CRISPR/Cas9-mediated therapeutics. Specifically, homology-directed replacement of aberrant genes, which is profoundly clinically applicable, is dependent upon utilizing HR-mediated DSB repair rather than NHEJ and is a major emphasis in modern molecular medicine. Compared to other proteins associated with NHEJ, Lig4 activity is predominantly restricted to NHEJ-mediated repair, making this target of high clinical value. As such, understanding the biological ramifications for decreased Lig4 activity is critical for informing future therapeutics not only in cancer biology, but across multiple disease states.

## Data Availability

TCGA data presented was from the METABRIC breast cancer dataset as described above. This data can be accessed through the TCGA data portal https://portal.gdc.cancer.gov or though cBioPortal http://www.cbioportal.org. The remainder of the data used to support the findings of this study are available from the corresponding author upon request.

## Supplementary Materials

Supplementary Materials
*Supplemental Figure 1: Doxorubicin did not augment cell death due to Lig4 depletion.* Lig4 depleted and control cells (NT) were subjected to the clonogenic assay following exposure to varying doses of doxorubicin (0.03, 0.1, 0.3, 1.0, 3.0, 10, 30, or 100 nM). Experiments were repeated three times.* Supplemental Figure 2: Lig4 knockdown does not enhance caspase activation.* (A, B) Levels of apoptotic markers cleaved caspase-3 and caspase-9 were assessed using western blot in Lig4 depleted and control cells treated with either replication toxins (1 *μ*M doxorubicin, 2 mM hydroxyurea, 10 *μ*M etoposide, 0.2 *μ*M camptothecin) or DMSO. Mean intensity values of cleaved caspase-3 or caspase-9 were first normalized to loading control, *β*-tubulin followed by a second normalization to vehicle treated control (NT) cells. Experiments were repeated four times and densitometry is presented with error bars representing the standard deviation.* Supplemental Figure 3*:* Lig4 depletion does not significantly exacerbate hydroxyurea-induced replication fork stalling.* (A, B) BT549 cells were treated with siRNA targeting either Lig4 or scrambled control (NT) and DNA replication was assessed using the DNA fiber assay. Briefly, cells were pulsed with thymidine analogs IdU and CldU separated by treatment with either PBS (A) or 10 mM hydroxyurea (HU; B). For each treatment, stalled, new and restarted replication forks were scored after blinding for treatment. Percent of stalled, new, and restarted replication forks in Lig4 depleted cells were compared with respective NT control cells (*∗*p<0.05,*∗∗*p<0.01, and *∗∗∗*p<0.001).* Supplemental Table 1: Copy number alterations in Lig4 are associated with increased Lig4 mRNA expression in basal but not nonbasal breast cancer patients.* mRNA expression z-scores generated using an Illumina Human v3 microarray by the Cancer Genome Atlas (TCGA) were retrieved using cBioPortal from patients with basal (n=209) or nonbasal (n=2300) breast cancer (BRCA). mRNA levels were stratified by copy number reported by TCGA and compared using a one-way ANOVA followed by Tukey's multiple comparisons test (n.s.= not significant, *∗*p<0.05, *∗∗*p<0.01, and *∗∗∗*p<0.001). Deep deletions were not reported in the basal BRCA dataset.* Supplemental Table 2: Statistical details regarding Figure *4. Each slide per treatment was visualized and images were captured from throughout the length of the slide. The number of images that were scored per treatment is shown in the table. The fibers were scored as red (stalled forks), green (new forks), or red + green (restarted forks). The total number of fibers scored for each treatment is listed. Stalled, new, and restarted forks are denoted as the percentage of total forks for each treatment condition.* Supplemental Table 3: Statistical details regarding Supplemental Figure 3*. Each slide per treatment was visualized and images were captured from throughout the length of the slide. The number of images that were scored per treatment is shown in the table. The fibers were scored as red (stalled forks), green (new forks), or red + green (restarted forks). The total number of fibers scored for each treatment is listed. Stalled, new, and restarted forks are denoted as the percentage of total scored forks for each treatment condition.* Supplemental Table 4: Statistical details regarding Figure *6.  Cells were visualized under 63X oil immersion and 10 images across per treatment were captured. *γ*H2AX foci were quantified using ImageJ software with DAPI stained area defined as region of interest. Non-DAPI stained region was used to account for background fluorescence and was subtracted from each of the *γ*H2AX mean intensity signal values. Mean intensity signal of *γ*H2AX was normalized to the DAPI stained area per image.
