# Supplementary material for: DNA Ligase IV Prevents Replication Fork Stalling and Promotes Cellular Proliferation in Triple Negative Breast Cancer
Source: J Nucleic Acids. 2019 Jan 31;2019:9170341. doi: 10.1155/2019/9170341 (PMC6374816; doi:10.1155/2019/9170341)
Supplement: Supplementary Materials — Supplemental Figure 1: Doxorubicin did not augment cell death due to Lig4 depletion. Lig4 depleted and control cells (NT) were subjected to the clonogenic assay following exposure to varying doses of doxorubicin (0.03, 0.1, 0.3, 1.0, 3.0, 10, 30, or 100 nM). Experiments were repeated three times. Supplemental Figure 2: Lig4 knockdown does not enhance caspase activation. (A, B) Levels of apoptotic markers cleaved caspase-3 and caspase-9 were assessed using western blot in Lig4 depleted and control cells treated with either replication toxins (1 μM doxorubicin, 2 mM hydroxyurea, 10 μM etoposide, 0.2 μM camptothecin) or DMSO. Mean intensity values of cleaved caspase-3 or caspase-9 were first normalized to loading control, β-tubulin followed by a second normalization to vehicle treated control (NT) cells. Experiments were repeated four times and densitometry is presented with error bars representing the standard deviation. Supplemental Figure 3: Lig4 depletion does not significantly exacerbate hydroxyurea-induced replication fork stalling. (A, B) BT549 cells were treated with siRNA targeting either Lig4 or scrambled control (NT) and DNA replication was assessed using the DNA fiber assay. Briefly, cells were pulsed with thymidine analogs IdU and CldU separated by treatment with either PBS (A) or 10 mM hydroxyurea (HU; B). For each treatment, stalled, new and restarted replication forks were scored after blinding for treatment. Percent of stalled, new, and restarted replication forks in Lig4 depleted cells were compared with respective NT control cells (∗p<0.05,∗∗p<0.01, and ∗∗∗p<0.001). Supplemental Table 1: Copy number alterations in Lig4 are associated with increased Lig4 mRNA expression in basal but not nonbasal breast cancer patients. mRNA expression z-scores generated using an Illumina Human v3 microarray by the Cancer Genome Atlas (TCGA) were retrieved using cBioPortal from patients with basal (n=209) or nonbasal (n=2300) breast cancer (BRCA). mRNA levels were stra [file 9170341.f1.pdf]

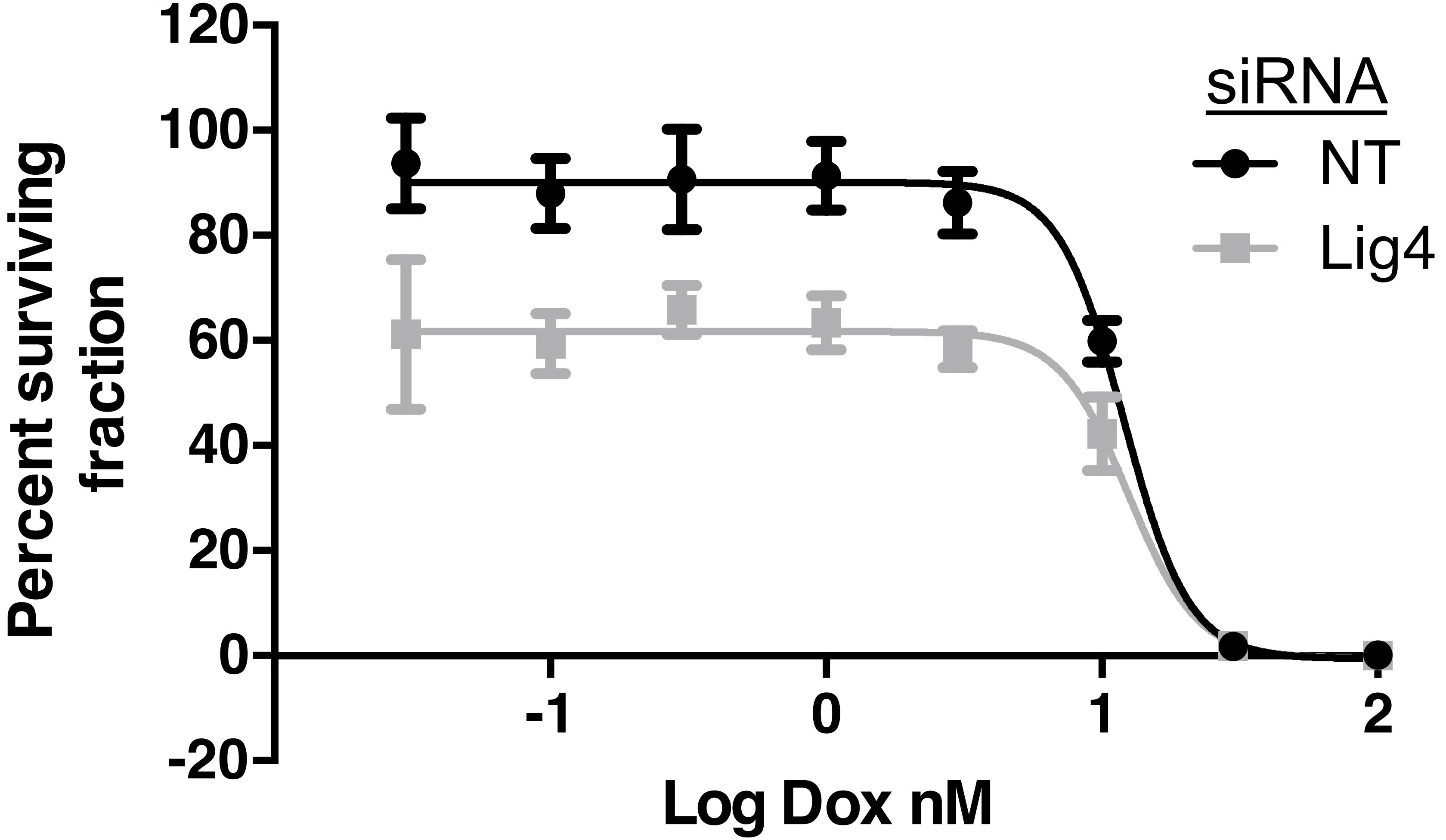

siRNA
NT
Lig4
120
100
80
60
40
20
0
-20
Percent surviving fraction
-1
0
1
2
Log Dox nM

Supplemental Figure 2.

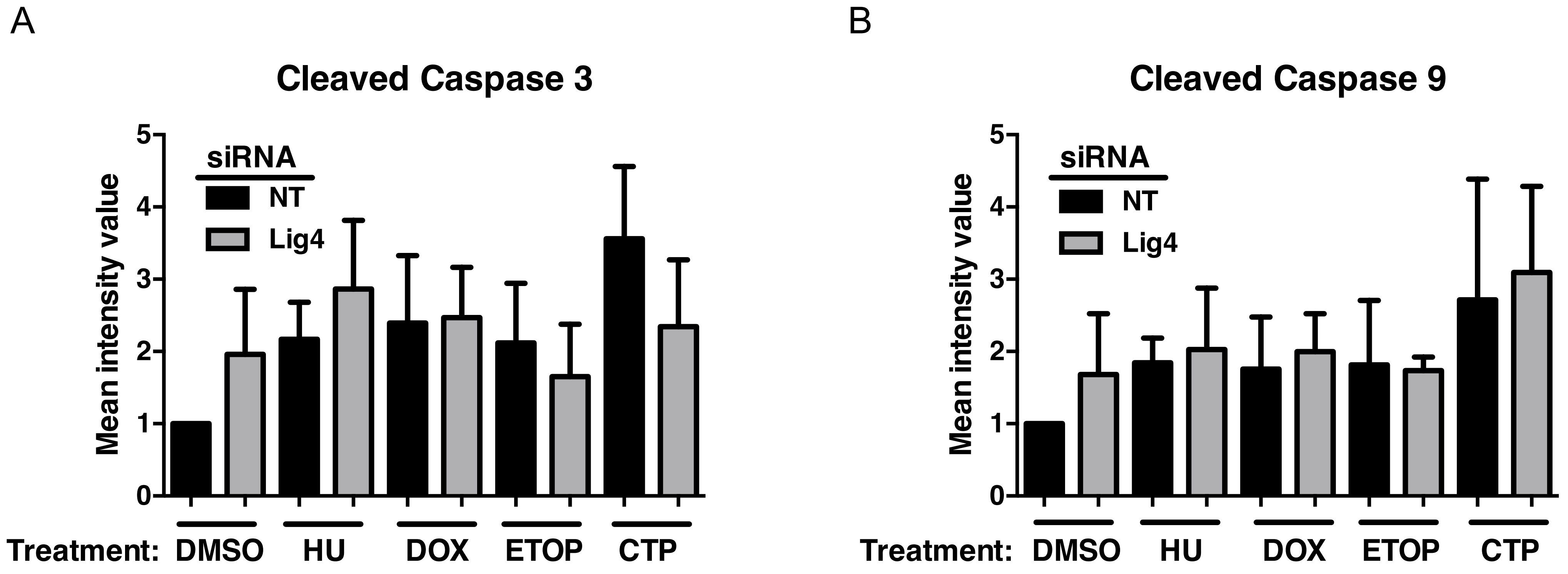

Supplementary Figure 3.

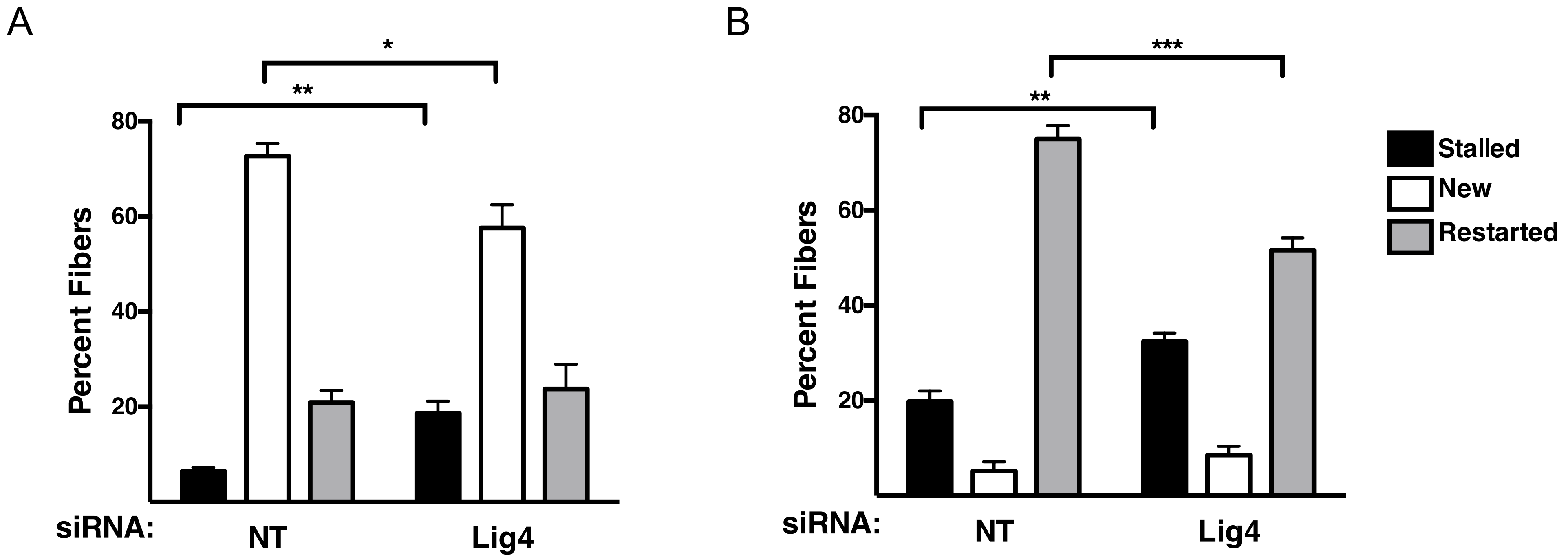

Supplemental Table 1.

Non-basal BRCA patient mRNA expression levels	Significant?	P value
Deep Deletion vs. Shallow Deletion	No	n.s.
Deep Deletion vs. Diploid	No	n.s.
Deep Deletion vs. Gain	No	n.s.
Deep Deletion vs. Amplified	No	n.s.
Shallow Deletion vs. Diploid	Yes	**
Shallow Deletion vs. Gain	No	n.s.
Shallow Deletion vs. Amplified	Yes	*
Diploid vs. Gain	No	n.s.
Diploid vs. Amplified	No	n.s.
Gain vs. Amplified	No	n.s.
**Basal BRCA patient mRNA expression levels**		
Shallow Deletion vs. Diploid	No	n.s.
Shallow Deletion vs. Gain	Yes	**
Shallow Deletion vs. Amplified	Yes	***
Diploid vs. Gain	Yes	**
Diploid vs. Amplified	Yes	***
Gain vs. Amplified	No	n.s.

Supplemental Table 2.

Treatment	NT siRNA	Lig4 siRNA
**Number of images scored**	16	28
**Number of fibers scored**	554	1344
**Total number of fibers scored**	1898	

Supplemental Table 3.

Treatment	NT siRNA + PBS	NT siRNA + HU	Lig4 siRNA + PBS	Lig4 siRNA + HU
**Number of images scored**	10	6	7	7
**Number of fibers scored**	725	424	649	531
**Total number of fibers scored**	2329			

Supplemental Table 4.

Treatment	NT siRNA	Lig4 siRNA
**Total number of nuclei scored**	85	53
